# Circulation of Non-Middle East Respiratory Syndrome (MERS) Coronaviruses in Imported Camels in Saudi Arabia

**DOI:** 10.7759/cureus.63351

**Published:** 2024-06-28

**Authors:** Yasser Alraddadi, Anwar Hashem, Esam Azhar, Ahmed Tolah

**Affiliations:** 1 Medical Microbiology and Parasitology, Faculty of Medicine, King Abdulaziz University, Jeddah, SAU; 2 Special Infectious Agent Unit, King Fahd Medical Research Center, King Abdulaziz University, Jeddah, SAU

**Keywords:** respiratory pathogens, camel imports, viral diversity, public health, zoonotic transmission, saudi arabia, dromedary camels, mers-cov, coronaviruses

## Abstract

Background

Coronaviruses (CoVs) pose significant health risks to humans, with recent outbreaks like severe acute respiratory syndrome coronavirus (SARS-CoV), Middle East respiratory syndrome coronavirus (MERS-CoV), and severe acute respiratory syndrome coronavirus 2 (SARS-CoV-2) underscoring their zoonotic potential. Dromedary camels (*Camelus dromedarius*) have been implicated as intermediate hosts for MERS-CoV, prompting heightened surveillance efforts. This study aims to identify non-MERS-CoV CoVs in imported camels at the Jeddah seaport, Saudi Arabia, using molecular techniques.

Methods

Camel nasal swabs (n = 337) were collected from imported dromedary camels arriving at the Jeddah Islamic seaport from Sudan and Djibouti. Samples were tested for CoVs using real-time real-time reverse transcription polymerase chain reaction (RT-PCR) targeting the RNA-dependent RNA polymerase gene. Positive samples were confirmed by conventional RT-PCR and Sanger sequencing. Selected samples underwent RNA sequencing to identify viral genomes. The study underscores the importance of molecular surveillance in camels to mitigate zoonotic risks.

Results

Out of 337 camel samples tested, 28 (8.30%) were positive for CoVs, predominantly from camels imported from Djibouti, compared to Sudan (13.39% vs. 5.78%). Sequence analysis confirmed the presence of non-MERS CoVs, including camel alpha-coronavirus and human CoV-229E-related strains. These findings highlight potential viral diversity and transmission risks in imported camel populations.

Conclusion

This study identifies diverse CoVs circulating in imported dromedary camels at the Jeddah Islamic seaport, Saudi Arabia, underscoring their potential role in zoonotic transmission. Enhanced surveillance and collaborative efforts are essential to mitigate public health risks associated with novel coronavirus strains from camel populations.

## Introduction

Understanding the zoonotic potential of various coronaviruses (CoVs) is crucial for anticipating and mitigating future outbreaks [1]. The emergence of severe acute respiratory syndrome coronavirus (SARS-CoV), Middle East respiratory syndrome coronavirus (MERS-CoV), and severe acute respiratory syndrome coronavirus 2 (SARS-CoV-2) highlights the importance of monitoring animal reservoirs for novel CoVs that threaten human health [2]. Identifying non-MERS-CoV CoVs in camels can provide essential insights into viral evolution and transmission dynamics, aiding in the development of effective public health strategies.

CoVs infect a variety of animals, causing respiratory, enteric, hepatic, and neurological diseases. These enveloped viruses with non-segmented, positive-sense RNA genomes belong to the order Nidovirales, specifically, the *Coronaviridae *family, which includes four genera: alpha, beta, gamma, and delta CoVs. CoVs pose significant health risks to humans and animals [3].

In humans, CoVs primarily infect the respiratory tract, causing illnesses from the common cold to fatal pneumonia. The first human CoVs, human coronavirus (HCoV)-OC43 and HCoV-229E, were identified in the 1960s [4]. From 2002 to 2003, SARS-CoV emerged in China, likely from bats, causing a global epidemic [4]. Subsequently, HCoV-NL63 and HCoV-HKU1 were discovered. In 2012, MERS-CoV emerged in Saudi Arabia, infecting nearly 2,562 people with a 34.4% mortality rate [5]. In December 2019, SARS-CoV-2 was identified in Wuhan, China, leading to the COVID-19 pandemic [6].

The World Health Organization (WHO) recorded 2,562 MERS-CoV cases, including 881 deaths, across 27 countries, with Saudi Arabia being the most affected [5]. Evidence supports MERS-CoV transmission between dromedary camels and humans, with camels likely serving as an intermediate host between bats and humans [7]. While camels are considered the primary source of human cases, many sporadic cases reported no direct contact with camels, with most secondary cases resulting from human-to-human transmission [5,7].

Imported camels from Africa enter Saudi Arabia via the Jeddah Seaport, which is linked to major trading routes. Recent studies have detected other CoVs in camels, such as human CoV-229E-related camel alpha-CoV and dromedary camel coronavirus UAE-HKU23 [8-10]. This suggests that camel importation could introduce new, divergent CoVs into Saudi Arabia, potentially leading to recombination events and increased viral diversity.

This study aimed to identify the presence of non-MERS-CoV in imported camels using molecular techniques. The findings could have significant implications for understanding CoV recombination in camels and the potential risks to human health, guiding both surveillance and intervention efforts to prevent future CoV outbreaks.

## Materials and methods

Camel samples

A total of 337 nasal swabs were collected from imported dromedary camels between August 2016 and August 2017. Samples were obtained from camels arriving via vessels from Sudan (n = 225) and Djibouti (n = 112) at Jeddah Islamic seaport before unloading. Nasal swabs were transported in refrigerated containers to King Fahd Medical Research Center (KFMRC), King Abdulaziz University, Jeddah, Saudi Arabia. Samples were stored in a viral transport medium (VTM) and kept on ice during transportation. Upon arrival, samples were aliquoted into pre-labeled cryotubes and stored at -80°C until RNA extraction.

Screening for CoVs

Viral RNA was extracted using the QIAamp Viral RNA mini kit (Qiagen, Germany) according to the manufacturer's instructions. Briefly, 140 µL of each nasal swab sample was lysed with 560 µL of lysis buffer, followed by incubation at room temperature for 10 minutes. After adding 560 µL of ethanol, the samples were washed twice with wash buffers and eluted in 60 µL of elution buffer. Extracted RNA was stored at -80°C until further analysis.

Extracted RNA was tested using real-time reverse transcription polymerase chain reaction (RT-PCR) with pan-CoV primers targeting a 440-bp fragment of the RNA-dependent RNA polymerase (RdRp) gene. The one-step SYBR Green RT-PCR kit (Qiagen, Germany) was used. The reaction mixture included 5 µL of RNA, 12.5 µL of 2X master mix, 0.25 µL of enzyme mix, and 1 µL of each primer, brought to a final volume of 25 µL with nuclease-free water. The cycling conditions were 50°C for 10 minutes, 95°C for five minutes, followed by 40 cycles of 95°C for 10 seconds and 50°C for 30 seconds, with a final extension at 72°C for one minute. Positive and negative controls were included in each run.

Confirmation of positive samples

Positive samples were confirmed using conventional one-step RT-PCR and Sanger sequencing. RT-PCR products were visualized on a 2% agarose gel, and bands of the expected size were extracted and purified using a DNA gel extraction kit (Norgen, Canada). Purified products were sequenced using an ABI 3500 Automatic Sequencer (Applied Biosystems, Waltham, MA, USA).

Genome sequencing

Selected positive samples were used for RNA sequencing. Libraries were constructed using the TruSeq Stranded Total RNA Gold kit (Illumina Inc., San Diego, CA, USA) and sequenced on an Illumina HiSeq 2500 platform, generating 150 bp reads. Paired-end reads were aligned to the dromedary camel genome using Bowtie V1.2.3. Unaligned reads with a quality score ≥20 were selected using the FASTX toolkit. These reads were then aligned against the complete RefSeq viral protein database using the DIAMOND aligner. Alignments with ≥75% sequence identity and an E-value of ≤10-10 were retained. Results were summarized and presented using Prism GraphPad V8.

## Results

Demographics of imported camels

A total of 337 nasal swab samples were collected from imported dromedaries between 2016 and 2017. The majority of samples were obtained from animals imported from Sudan (n = 225, 66.77%) compared to Djibouti (n = 112, 33.23%). Most animals were older than two years (64.99%), and males comprised 59.35% of the tested animals (n = 200) compared to females (n = 137, 40.65%) (Table 1).

**Table 1:** Demographics of camels used in this study (n = 337) Data are presented as frequency (n) and percentage (%).

Variable		Frequency	Percentage
Age	<2 years	118	35.01%
	>2 years	219	64.99%
Country of origin	Sudan	225	66.77%
	Djibouti	112	33.23%
Gender	Male	200	59.35%
	Female	137	40.65%

Detection of CoVs

Out of the 337 samples tested by real-time RT-PCR, 28 samples (8.30%) were positive for CoVs. The detection rate was significantly higher in animals older than two years compared to those younger than two years (10.96% vs. 3.92%, p = 0.025). Additionally, significantly more positive samples were detected in animals imported from Djibouti compared to Sudan (13.39% vs. 5.78%, p = 0.029). There was no significant difference in CoV positivity between male (9.50%) and female (6.57%) animals (Table 2). The cycle threshold (Ct) values ranged from 34.66 to 39.80, indicating low viral loads (Table 3).

**Table 2:** Detection rate of CoVs in imported dromedary camels in Saudi Arabia Data are presented as frequency (n) and percentage (%). Statistical significance was determined using the chi-square test, with p < 0.05 considered significant. CoVs, coronaviruses

Variable		Total number	Positive cases, n (%)	p-value
Age	<2 years	118	4 (3.92%)	0.025
	>2 years	219	24 (10.96%)	
Country of origin	Sudan	225	13 (5.78%)	0.029
	Djibouti	112	15 (13.39%)	
Gender	Male	200	19 (9.50%)	0.377
	Female	137	9 (6.57%)	
Total		337	28 (8.30%)	N/A

Confirmation of CoV presence

Positive samples were confirmed using conventional RT-PCR and Sanger sequencing, showing bands of expected size on agarose gel and high sequence homology (~98-99%) with alpha CoVs such as Camel229E-CoV. High-depth RNA sequencing revealed positive hits to CoVs in four out of 32 samples, predominantly belonging to camel alphacoronavirus.

Next generation metagenomics

**Figure 1:**
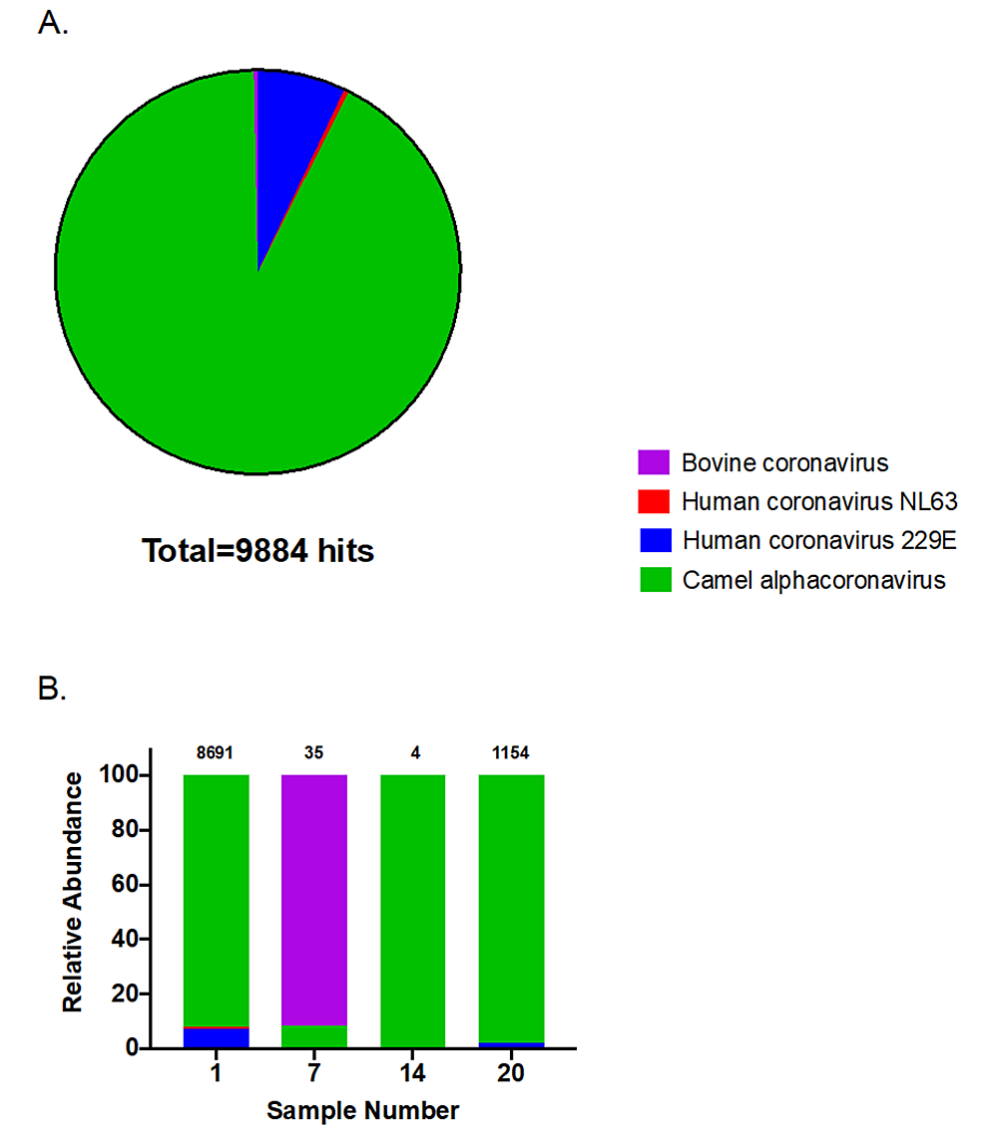
Identified coronavirus genomes (A) Combined hits for all samples; (B) individual samples. The number on top of each bar indicates the total number of coronavirus hits per sample.

**Figure 2:**
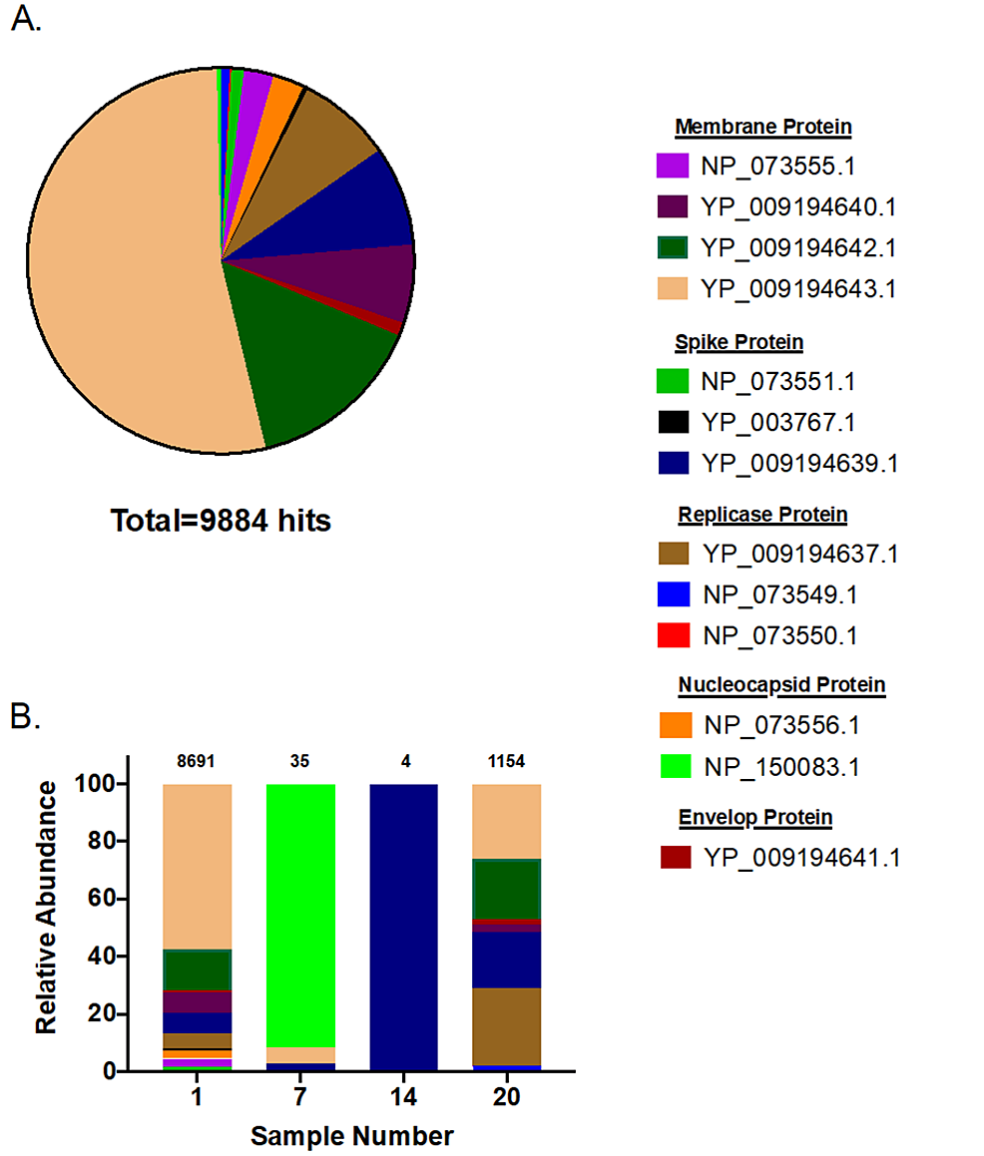
Annotation of coronavirus hits (A) Combined hits for all samples; (B) individual samples. The number on top of each bar indicates the total number of coronavirus hits per sample.

In order to recover coronavirus from camel samples, high-depth RNA sequencing was performed. The average number of high-quality reads was 20,949,686 ± 15,943,548 (SD), ranging from 659,093 to 62,518,785 high-quality reads per sample. Aligning reads belonging to the 32 camel samples against the viral RefSeq proteins database showed only four samples (samples 1, 7, 14, and 20) with positive hits to coronavirus. The total number of coronavirus hits for sample 1 is 8,691, for sample 7 is 35, for sample 14 is 4, and for sample 20 is 1,154 (total number of hits = 9,884). The majority of hits (92.2%) belonged to camel alphacoronavirus, and the reaming hits belonged to HCoV-229E (7%), HCoV-NL63 (0.4%), and bovine coronavirus (0.4%) (Figure 1). Hits to camel alphacoronavirus were predominant for samples 1 (92%), 14 (100%), and 20 (97.7%), while Bovine coronavirus hits dominated reads obtained from sample 7 (91.4%). The annotation of the obtained hits was mainly attributed to the coronavirus membrane protein (77.4%), followed by spike protein (9.9%), replicase polyprotein (8.6%), nucleocapsid protein (3%), and envelope protein (1.1%). A high proportion of coronavirus reads from sample 1 belonged to the membrane protein YP_009194643.1 (57.3%), while almost all hits for sample 7 belonged to the nucleocapsid protein NP_150083.1 (91.5%). On the other hand, all coronavirus reads from sample 14 (n = 4) were aligned to the spike protein YP_009194639.1. Finally, hits from sample 20 were more versatile and belonged mainly to the replicase polyprotein YP_009194637.1 (26.7%), the membrane proteins YP_009194643.1 (26%) and YP_009194642.1 (21%), and the spike protein YP_009194639.1 (19.4%) (Figure 2).

## Discussion

Emerging and re-emerging pathogens pose significant global health threats, often originating from animal reservoirs and potentially leading to pandemics with profound implications for public health and the economy. Early detection, prediction, and risk assessment of pathogens in animal populations are crucial components of a One Health approach, essential for preparedness and mitigating their potential spread to humans [11].

MERS is caused by MERS-CoV and emerged in Saudi Arabia in 2012, resulting in approximately 2,500 laboratory-confirmed cases and around 850 deaths reported to the WHO since then [12]. Unlike SARS-CoV, which likely originated from a single spillover event, MERS-CoV has demonstrated multiple introductions into humans from animal reservoirs [13]. Dromedary camels have been identified as the natural reservoir of MERS-CoV since 1992 in the Middle East and 1983 in Africa, highlighting their role in the virus's ecology [13-15]. The discovery of CoVs related to MERS-CoV in African bats has led to the hypothesis that these viruses may have been transmitted to camels in Africa, subsequently spreading to the Middle East through camel trade [15].

In camels, MERS-CoV infection typically manifests with mild or no respiratory symptoms, complicating early detection efforts [14]. Surveillance studies in dromedary camels are therefore essential for monitoring the circulation of MERS-CoV and other CoVs using appropriate laboratory methods. Despite Saudi Arabia's large camel population and significant annual camel imports from Africa, comprehensive surveillance systems are currently lacking, posing potential public health risks. Continuous surveillance is critical not only for detecting MERS-CoV but also for identifying other CoVs in camels and monitoring potential recombination events leading to the emergence of novel CoV strains.

Metagenomic sequencing analyses of MERS-CoV-positive dromedary camels in Abu Dhabi have revealed the presence of various viral sequences, underscoring the potential for co-circulation and recombination of CoVs in camels [16]. While dromedaries are the primary reservoir, Bactrian camels, found in Central Asia, have not shown evidence of MERS-CoV infection based on current studies. Studies have reported high seroprevalence of MERS-CoV in dromedaries across the Middle East and Central Africa [13,16], yet the presence of MERS-CoV in Bactrian camels remains inconclusive.

In our study, 28 out of 337 nasal swab samples (10.98%) tested positive for CoVs by RT-PCR, with subsequent sequencing confirming the presence of non-MERS-CoV sequences. Detected genomes included sequences related to human CoV-229E and NL63, camel alpha-CoV, and bovine CoVs, suggesting camels may serve as mixing vessels for CoVs.

Continuous surveillance of CoVs in animals, particularly camels, is imperative for developing effective strategies to control their spread. Hygiene practices among farmers, herdsmen, and other animal handlers are crucial in minimizing the transmission of CoVs to humans and other animals. Although there are no reported human infections with CoVs other than MERS-CoV from camel contact, the potential for interspecies transmission, recombination, and co-infections in camels warrants further investigation.

This study provides molecular evidence of several CoVs circulating in dromedary camels in Saudi Arabia, indicating their potential role in zoonotic transmission to other livestock and humans. These findings highlight the critical need for enhanced field surveillance of CoVs and other pathogens in dromedary camels to implement effective preventive strategies. Collaboration between the Ministry of Environment, Water, and Agriculture and the Ministry of Health is essential for developing and implementing robust preventive plans and programs aimed at mitigating CoV spread from camels to humans. Additionally, stringent governmental policies are necessary to thoroughly investigate and screen imported camels for contagious pathogens, thus minimizing the risk of disease transmission. Overall, proactive surveillance and collaborative efforts among governmental bodies are crucial for safeguarding public health against potential outbreaks of CoVs and other emerging infectious diseases originating from camel populations.

## Conclusions

This study provides molecular evidence of several CoVs circulating in dromedary camels in Saudi Arabia, indicating their potential role in zoonotic transmission to other livestock and humans. These findings highlight the critical need for enhanced field surveillance of CoVs and other pathogens in dromedary camels to implement effective preventive strategies. Collaboration between the Ministry of Environment, Water, and Agriculture and the Ministry of Health is essential for developing and implementing robust preventive plans and programs aimed at mitigating CoV spread from camels to humans. Additionally, stringent governmental policies are necessary to thoroughly investigate and screen imported camels for contagious pathogens, thus minimizing the risk of disease transmission. Overall, proactive surveillance and collaborative efforts among governmental bodies are crucial for safeguarding public health against potential outbreaks of CoVs and other emerging infectious diseases originating from camel populations.
